# Analysis of the Mechanisms of Rice *oszfp30* Mutant in Improving Forage-Quality-Related Traits Based on Transcriptome and Metabolome Analyses

**DOI:** 10.3390/genes17091086

**Published:** 2026-09-09

**Authors:** Chenfei Dong, Ruijie Zhao, Pengtao Shang, Yichen Jiang, Yue He, Yanhong Yan, Chao Chen

**Affiliations:** 1 College of Landscape Architecture, Jiangsu Vocational College of Agriculture and Forestry, Jurong 212400, China; 2025220007@njau.stu.edu.cn (C.D.);; 2 College of Animal Science, Guizhou University, Guiyang 550025, China; 3 College of Grassland Science and Technology, Sichuan Agricultural University, Chengdu 611130, China

**Keywords:** *oszfp30* mutant, transcriptome, metabolome, forage quality, cellulose, hemicellulose, lignin

## Abstract

Background/Objectives: Using straw as feed is crucial for addressing resource scarcity and waste-based environmental pollution and alleviating the contradiction between forage production and food security. However, rice straw has disadvantages such as a high lignin, cellulose, and hemicellulose content, as well as a dense cell wall structure, which greatly limit its utilization as feed. Improving the forage quality of rice straw through genetic breeding is an important means of enhancing its utilization rate. Methods: To explore the molecular basis underlying the improved nutritional and cell wall compositional traits of the rice lines (*oszfp30-1* and *oszfp30-2*) obtained in previous studies, we conducted phenotypic, physiological, transcriptomic, and metabolomic analyses on *oszfp30-1*, *oszfp30-2* and the wild type (WT) after 50 days of pot cultivation. Results: *oszfp30-1* and *oszfp30-2* exhibited higher plant height, above-ground biomass, crude protein, and crude fat content, while their hemicellulose, cellulose, and lignin contents were significantly lower than those of the wild type. Transcriptomics was used to identify 737 common differentially expressed genes (DEGs), and metabolomics was used to identify 189 common differentially expressed metabolites (DEMs). KEGG analysis revealed that these DEGs and DEMs were significantly enriched in terpene biosynthesis, starch and sucrose metabolism, phenylpropanoid biosynthesis, and amino sugar and nucleotide sugar metabolism. Conclusions: Our research reveals that the improvement in forage-quality-related traits is closely related to key genes involved in gibberellin synthesis, including *GA20ox*, *GA2ox*, and *GA3ox*; key genes involved in cellulose synthesis, including *OsSUS* and *UGPase*; key genes involved in hemicellulose synthesis, including *UXS* and *IRX10*; and key genes involved in lignin synthesis, including *CCR*, *CAD*, *CCoAOMT*, and *COMT*. These identified DEGs and DEMs are associated with the *OsZFP30* mutation and may be downstream targets of this transcription factor, providing a foundation for further research on the *OsZFP30* regulatory network.

## 1. Introduction

As the global population grows and the demand for animal protein increases, the issue of feed shortage is becoming increasingly severe. Rice (*Oryza sativa* L.) is the staple food for over half of the world’s population, and generates hundreds of millions of tons of straw annually [1]. Traditionally, rice straw has often been burned or discarded, resulting in not only resource waste but also severe environmental issues. Utilizing rice straw as feed and developing it into a high-quality product suitable for livestock represents an important strategy to alleviate the competition between humans and livestock for grain resources and to promote sustainable agricultural development [2]. The feeding value of rice straw is primarily determined by its nutritional components (such as crude protein content) and digestibility, which are directly constrained by the structure and chemical composition of the cell wall, and especially the content and cross-linking degree of cellulose, hemicellulose, and lignin [3].

A high lignin content and cellulose crystallinity significantly reduce the in vitro degradation rate of straw and its intake and digestibility by animals [4]. Currently, physical, chemical, and biological pretreatment methods are commonly used to improve the digestibility and degradation rate of rice straw, but their improvement effects are often unsatisfactory and the costs are high [5]. Therefore, reducing the lignin content of straw and optimizing its cellulose structure through molecular breeding techniques, while ensuring the normal growth of rice, is an important approach to addressing the challenge of utilizing rice straw as feed. Under normal circumstances, reducing the content of cell wall fiber substances can enhance digestibility, but it can also lead to decreased stem hardness and reduced lodging resistance in rice, subsequently reducing grain yield and causing other negative outcomes [6]. Therefore, identifying key genes that can both maintain normal rice growth and enhance the digestibility and degradation rate of rice straw fiber substances is crucial for improving the forage quality of rice straw through molecular breeding methods.

Transcription factors are a class of proteins that can bind to the promoter regions of genes, promoting or inhibiting the binding of RNA polymerase to the promoter and thereby positively or negatively regulating the expression of target genes [7]. Researchers have identified dozens of transcription factors to date that regulate the synthesis of cell wall polymers, such as NACs and MYBs [8,9]. These transcription factors exert their function in regulating the degradation rate of the cell wall by activating or inhibiting the expression of genes involved in fiber-like substance synthesis or by binding to the cell wall [10]. NAC transcription factors are unique to plants, and some of them are related to the formation of secondary walls [11]. The lack of function of secondary-wall-related NAC domain protein 1 (snd1) leads to abnormal secondary walls and ectopic deposition [12]. Secondary-wall-related NAC transcription factors (SWN) regulate the biosynthesis of the secondary wall, but the mechanism underlying the deposition of cellulose, hemicellulose, and lignin remains unclear [13]. MYB transcription factors are highly conserved and possess one to three incomplete repeats of amino acid sequences (R1, R2, and R3). It has been reported that MYB can regulate the formation of secondary walls in *Arabidopsis thaliana* [14]. Recent studies have found that the plant height and lodging resistance of *Osmyb110* rice mutants are significantly enhanced, but further research is needed to determine whether MYB110 regulates the synthesis of secondary cell walls or fibrous substances [15]. Furthermore, site-specific mutations in MYB transcription factors can alter the composition of rice cell walls, not only maintaining normal plant growth but also enhancing the digestibility of rice straw [11]. In summary, it is feasible to achieve a balance between normal rice growth and improved straw digestibility by regulating specific transcription factors.

Our preliminary research found that exogenous gibberellin (GA) treatment has a significant effect on the fiber composition and content of rice straw in different types of rice [16], and has a positive effect on rice production [17]. The transcriptome results showed that the expression of *OsZFP30*, a transcription factor encoding RanBP2 zinc finger protein, was regulated by GA, and its expression level was significantly correlated with the content of rice straw fiber-like substances, suggesting that it may directly participate in regulating their synthesis. Previous studies have shown that RanBP2-type zinc finger transcription factors play a role in leaf size, stem elongation, and secondary wall thickening [18,19]. However, the functions and mechanisms of *OsZFP30* in regulating the synthesis of straw fiber-like substances, as well as digestibility, and forage quality, have not yet been reported. Therefore, in this study, transgenic rice lines were generated and investigated at the transcriptional, metabolic, and physiological levels to lay a foundation for further dissecting the function of *OsZFP30*.

## 2. Materials and Methods

### 2.1. Plant Materials

The rice material used in this study was the japonica rice variety Zhonghua 11 (Wild Type, WT) and its mutants generated via CRISPR/Cas9 technology. The mutant lines carried a mutation in the *OsZFP30* gene (for specific information about the *Oszfp30* mutant, please refer to the Figures S1–S3) and were previously preserved by Jiangsu Agriculture and Forestry Vocational and Technical College.

### 2.2. Experimental Design

We selected 50 plump and uniform seeds of the wild type and the mutants (*oszfp30-1* and *oszfp30-2*), respectively, and removed the glumes before disinfection. The disinfection procedure was as follows: the seeds were placed in 50 mL centrifuge tubes, soaked in 70% ethanol for 1 min, and rinsed three times with sterile water; they were then soaked in 2.5% sodium hypochlorite (NaClO) solution for 15 min with intermittent shaking; finally, the disinfectant was discarded, and the seeds were rinsed five to six times with sterile water, each time for 1–2 min. The sterilized seeds were evenly placed in Petri dishes (9 cm in diameter) lined with two layers of filter paper, and an appropriate amount of sterile water was added to moisten the filter paper. The dishes were placed in an incubator at 28 °C in the dark to germinate. An appropriate amount of sterile water was added daily to maintain moisture. When the radicle elongated to approximately 2–3 cm and the plumule emerged from the seed coat (after about 3–5 days), seedlings of uniform growth were selected for transplanting.

Uniformly grown rice seedlings were transplanted into plastic pots (20 cm in diameter, 15 cm in height), with one seedling per pot. The cultivation substrate was a mixture of garden soil and vermiculite at a volume ratio of 2:1. After transplanting, the seedlings were placed in an artificial climate chamber under the following conditions: photoperiod of 14 h light/10 h dark, light intensity of 800 μmol·m^−2^·s^−1^, day/night temperature of 28 °C/24 °C, and relative humidity of 70–80%. The soil was kept moist throughout the growth period. The pots were arranged in a completely randomized design in the artificial climate chamber, and the plant positions were randomly reassigned weekly to minimize positional effects. The leaves and stems of each rice plant sample were collected and measured for indicators on the 50th day after transplanting. For phenotypic, physiological index determination, and transcriptomics analysis, each genotype was set up with 3 biological replicates, with each replicate consisting of 6 independent plants. For metabolomics analysis, each genotype was set up with 6 biological replicates, with each replicate consisting of 6 independent plants. Each replicate was made up of equal tissue samples collected from 6 randomly selected plants to reduce inter-individual variation.

### 2.3. Determination of Physiological Indicators

The above-ground parts of rice plants were collected, treated at 105 °C for 0.5 h, and then dried at 70 °C to a constant weight. The dry weight was recorded as above-ground biomass. The cellulose, hemicellulose, and lignin contents were determined using reagent kits (BC4280, BC4440, and BC4200; Solarbio, Beijing, China) according to the manufacturer’s instructions [20]. Crude protein (CP) content was determined according to Vadiveloo’s method [21], and ether extract (EE) content was determined according to Hossain’s method [22].

### 2.4. Widely Targeted Metabolomic Analysis

Following vacuum freeze-drying in a Scientz-100F freeze dryer (Ningbo Scientz Biotechnology Co., Ltd., Ningbo, China), each sample (100 mg) was accurately weighed and finely ground to a homogeneous powder. The resulting powder was reconstituted in 1.2 mL of 70% (*v*/*v*) methanol and subjected to repeated vortexing to facilitate metabolite extraction. Subsequently, the mixture was centrifuged at 12,000 rpm for 10 min at 4 °C, and the supernatant was passed through a 0.22 μm microporous filter membrane. The filtered extracts were transferred to sample vials and kept ready for UPLC-MS/MS analysis.

Chromatographic separation was carried out under the following conditions: an Agilent SB-C18 column (1.8 µm, 2.1 mm × 100 mm) was employed; the mobile phase consisted of ultrapure water supplemented with 0.1% formic acid (aqueous phase A) and acetonitrile supplemented with 0.1% formic acid (organic phase B). A gradient elution program was applied as follows: 5% B at 0 min, 20% B at 2 min, 25% B at 4 min, 60% B at 9 min, 100% B at 14 min, 100% B at 18 min, 5% B at 18.1 min, and 5% B at 19.5 min. The flow rate, column temperature, and injection volume were maintained at 0.35 mL·min^−1^, 40 °C, and 4 μL, respectively.

Upon elution from the column, the analytes were introduced into an electrospray ionization (ESI) triple quadrupole (QQQ) mass spectrometer operated in alternating positive and negative ion modes. The key MS parameters were configured as follows: the ion source temperature was set to 550 °C for both positive and negative modes; the ion spray voltage was 5.5 kV in positive mode and 4.5 kV in negative mode; ion source gas I, gas II, and curtain gas (CUR) were adjusted to 50 psi, 60 psi, and 25 psi, respectively; and the declustering potential was 100 V for positive mode and −100 V for negative mode. Collision-induced dissociation (CID) parameters were set at relatively optimized high values. Within the triple quadrupole, each ion pair was monitored and detected using individually optimized declustering potential and collision energy.

Pearson correlation coefficients (PCCs) across all samples were computed using R-4.0.3 software, and inter-sample relationships were visualized as a heatmap generated with the PheatMap package in R [23]. Multivariate statistical analyses, encompassing principal component analysis (PCA), partial least squares discriminant analysis (PLS-DA), and orthogonal partial least squares discriminant analysis (OPLS-DA), were performed on the preprocessed data using SIMCA-P 13.0. The PLS-DA model was validated using 200 permutation tests [24]. Differentially expressed metabolites (DEMs) were screened based on Variable Importance in Projection (VIP) ≥ 1 from the OPLS-DA model, |log_2_(fold change)| ≥ 1, and *p*-value < 0.05 (Student’s *t*-test). The VIP value incorporates multivariate information and serves as a multiple-testing-aware filter in the metabolomics field; the univariate *p*-value was used as an additional criterion. *p*-values were further adjusted using the Benjamini–Hochberg method, and DEMs were required to meet adjusted *p*-value < 0.05. To elucidate the biological pathways associated with these DEMs, functional annotation and pathway enrichment analysis were conducted using the KEGG database.

### 2.5. Transcriptomic Analysis

Total RNA was isolated from wild-type plants and the two mutant lines (oszfp30-1 and oszfp30-2) with the TRIzol reagent kit. Following purification, RNA purity was determined on a NanoDrop One spectrophotometer (Thermo Fisher Scientific, Waltham, MA, USA), while RNA integrity was verified using a Bioanalyzer 2100 (Agilent, Santa Clara, CA, USA). mRNA was subsequently enriched and purified using the VAHTS mRNA Capture Beads kit (Vazyme Biotech Co., Ltd., Nanjing, China), and cDNA libraries were prepared with the VAHTS Universal V8 RNA-seq Library Prep Kit (Vazyme Biotech Co., Ltd., Nanjing, China) for Illumina. The quality of the constructed libraries was checked using Qubit 4.0, and high-throughput sequencing was conducted on the DNBSEQ-T7 platform in paired-end (PE) mode.

Raw sequencing reads were preprocessed with fastp v0.23.2 [25] to remove adapter-contaminated reads, low-quality reads, and reads with an unknown base (N) content exceeding 5%, thereby yielding high-quality clean reads. The clean reads were then mapped to the reference genome (Genome assembly AGIS1.0, GCF_034140825.1) using STAR v2.7.10a. Transcriptome assembly was performed using Trinity, followed by clustering and redundancy removal using Tgicl to generate Unigenes. Functional annotation of the Unigenes was carried out using DIAMOND against the KEGG, NR, Swiss-Prot, GO, KOG, and Trembl databases [26].

Gene expression differences were analyzed using DESeq2, and genes meeting the criteria of adjusted *p*-value (padj) < 0.05 and |log_2_(fold change)| > 1 were classified as differentially expressed genes (DEGs). The Benjamini–Hochberg procedure was applied for multiple testing correction [27]. To characterize the principal biological functions and regulatory pathways associated with these DEGs, Gene Ontology (GO) enrichment analysis was implemented in TBtools (v2.362), while Kyoto Encyclopedia of Genes and Genomes (KEGG) pathway enrichment analysis was performed using the KEGG database [28].

### 2.6. qRT-PCR Analysis

To confirm the reliability of the RNA-seq results, qRT-PCR was performed using the same total RNA preparations employed for transcriptome sequencing. Ten representative DEGs (*GA20ox1*, *GA20ox2*, *GA3ox1*, *GA3ox2*, *OsSUS1*, *UGPase2*, *UXS1*, *IRX10*, *CCR1*, and *CAD1*) were chosen for validation. First-strand cDNA was synthesized from total RNA via reverse transcription using the TransScript II cDNA Synthesis SuperMix kit (TransGen Biotech, Beijing, China). Three independent RNA extractions were included as biological replicates to guarantee result reproducibility. Gene-specific primers were designed with Primer-BLAST (NCBI) https://www.ncbi.nlm.nih.gov/tools/primer-blast/index.cgi?LINK_LOC=BlastHome, and *OsACTIN1* served as the endogenous reference gene. Relative expression levels were quantified using the comparative CT (2^−ΔΔCT^) method [29].

### 2.7. Statistical Analysis

Data compilation and preliminary calculations were performed in Microsoft Excel 2021. Statistical comparisons were conducted via one-way ANOVA followed by Duncan’s multiple range test using SPSS Statistics 20.0 (SPSS Inc., Chicago, IL, USA). All graphical representations were produced with Origin 2021 software.

## 3. Results

### 3.1. Phenotype Analysis of Strain

**Figure 1:**
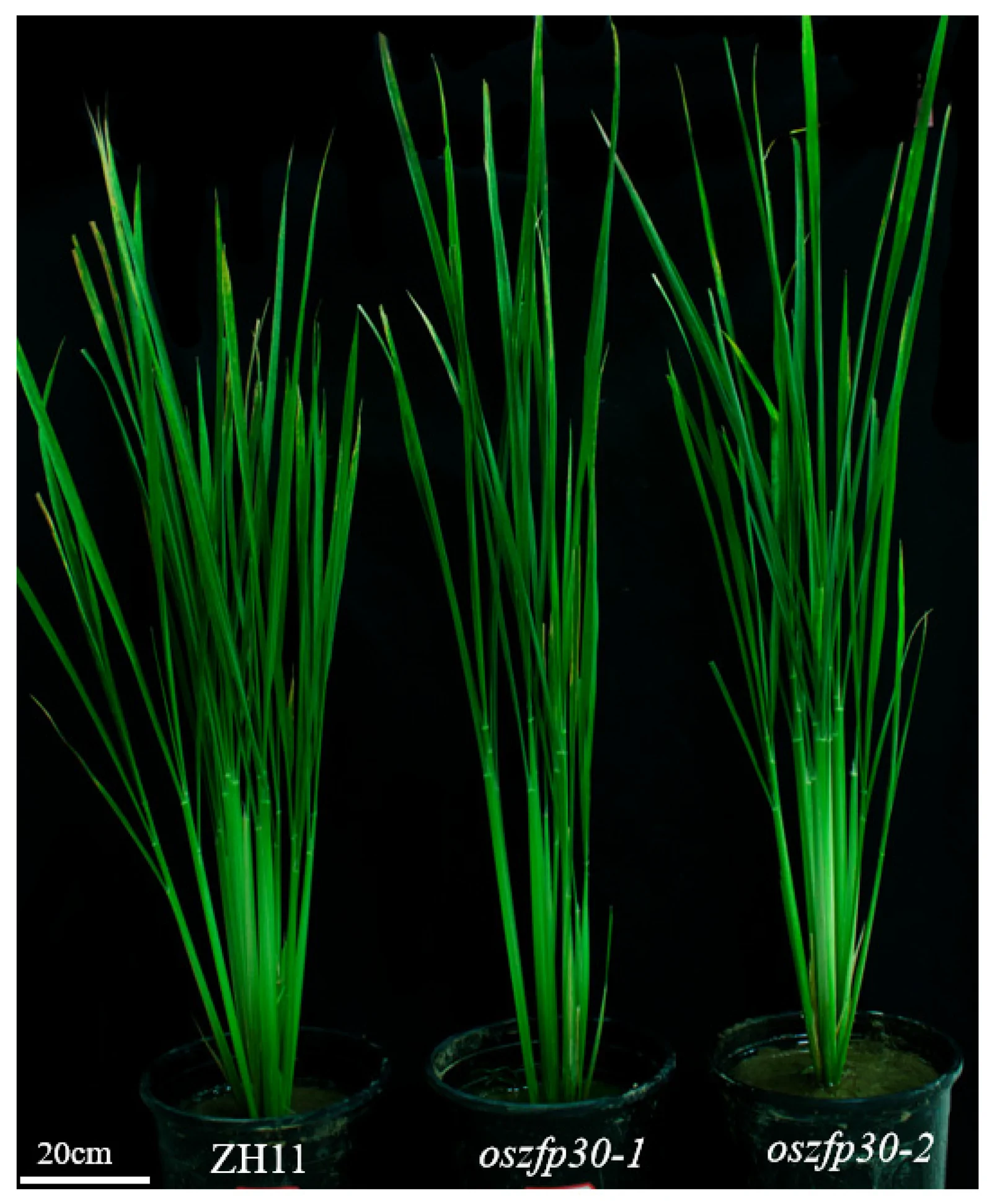
Phenotype comparison between lines and wild-type rice.

Compared with the wild type, the plant height and above-ground biomass of strains *oszfp30-1* and *oszfp30-2* were significantly higher (Figure 1 and Table 1), although their tiller numbers did not change significantly (Table 1). These results indicate that mutations in the *OsZFP30* gene do not affect the main agronomic traits of rice and show favorable changes in biomass production that may benefit the forage value of rice straw.

### 3.2. Fiber Components of Strain Content

**Figure 2:**
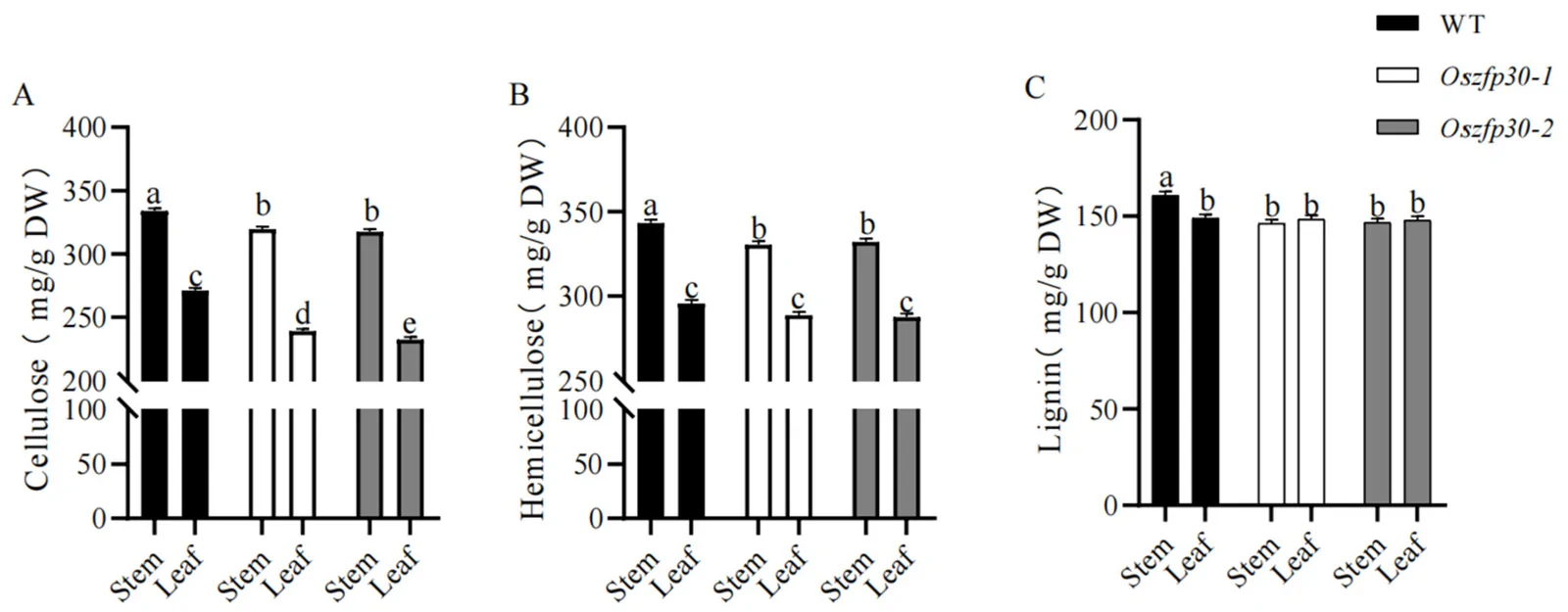
Fiber component contents of lines and wild-type rice. (**A**). Cellulose content; (**B**). Hemicellulose content; (**C**). Lignin content. Note: Different lowercase letters after the same column of data indicate significant differences (*p* < 0.05); the data is formatted as mean ± standard deviation.

The cellulose, hemicellulose, and lignin contents in the stem and leaf tissues of wild-type (WT) and transgenic lines (*oszfp30-1* and *oszfp30-2*) are presented in Figure 2. In stems, WT had significantly higher cellulose, lignin and hemicellulose contents than *oszfp30-1* and *oszfp30-2*. In leaves, WT also showed significantly higher cellulose and hemicellulose contents than the transgenic lines, whereas no significant differences in lignin content were observed among the three genotypes.

### 3.3. Nutritional Components of Strains

**Figure 3:**
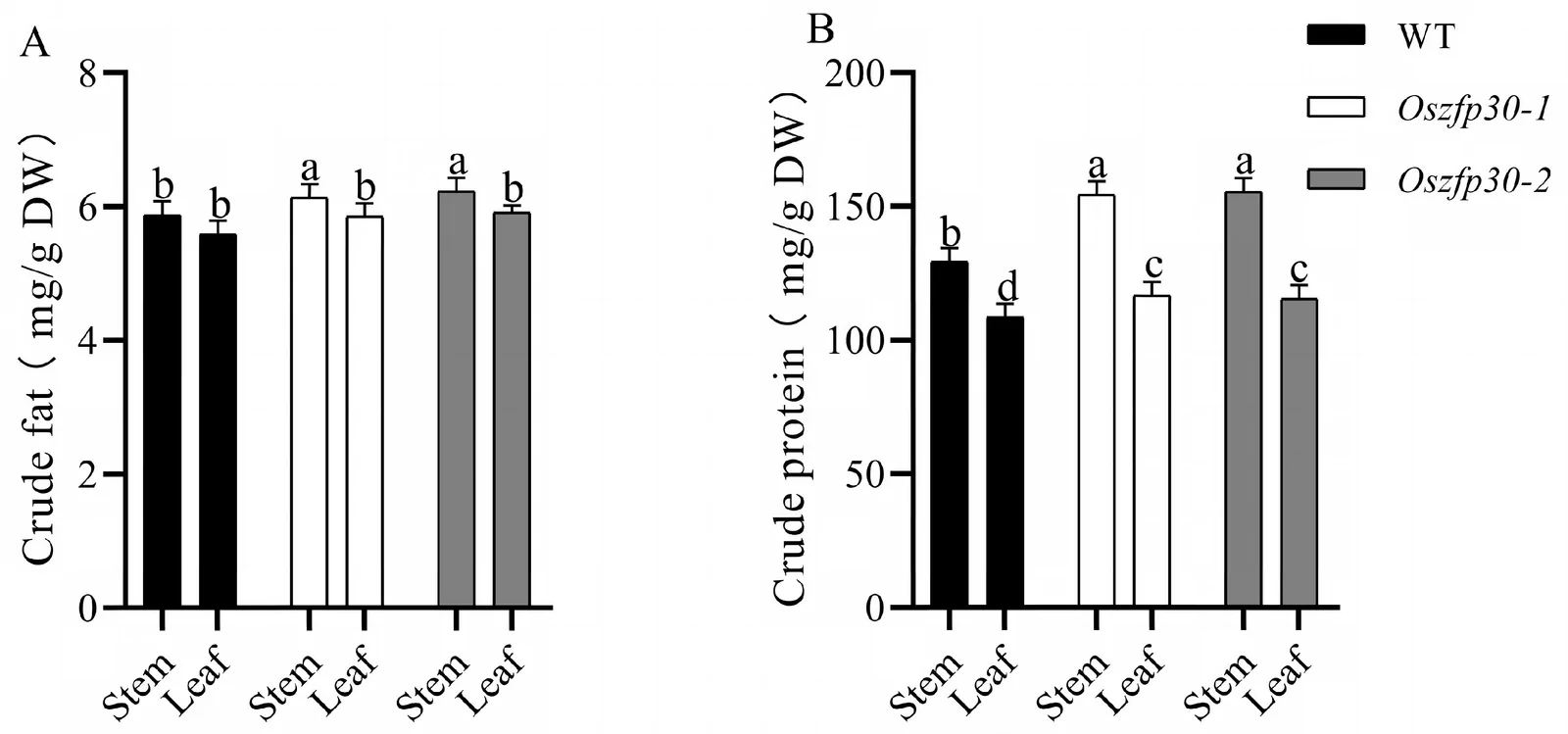
Nutritional components of strain and wild-type rice. (**A**). Crude fat content; (**B**). Crude protein content. Note: Different lowercase letters after the same column of data indicate significant differences (*p* < 0.05); the data is formatted as mean ± standard deviation.

The crude fat and crude protein contents in WT and transgenic lines (*oszfp30-1* and *oszfp30-2*) are shown in Figure 3. In stems, both *oszfp30-1* and *oszfp30-2* had significantly higher crude fat and crude protein contents than WT. In leaves, crude fat content did not differ among the three genotypes, whereas crude protein content was significantly higher in the transgenic lines than in WT.

### 3.4. Statistical Analysis of Transcriptome Sequencing Data

In this transcriptome analysis, a total of nine cDNA libraries were constructed and sequenced to obtain raw data. The GC content of the raw data from the nine samples exceeded 48%, with Q20 values all greater than 98% and Q30 values all greater than 96% (Table 2), indicating that the transcriptome sequencing quality was good and met the requirements for data analysis. The clean data were aligned to the reference genome, Genome assembly AGIS1.0 (GCF_034140825.1), and the alignment rate for each sample reached over 95% (Table 2), indicating that the alignment results for the selected reference genome were normal and met the requirements for gene functional annotation and analysis.

### 3.5. Identification of Differentially Expressed Genes

**Figure 4:**
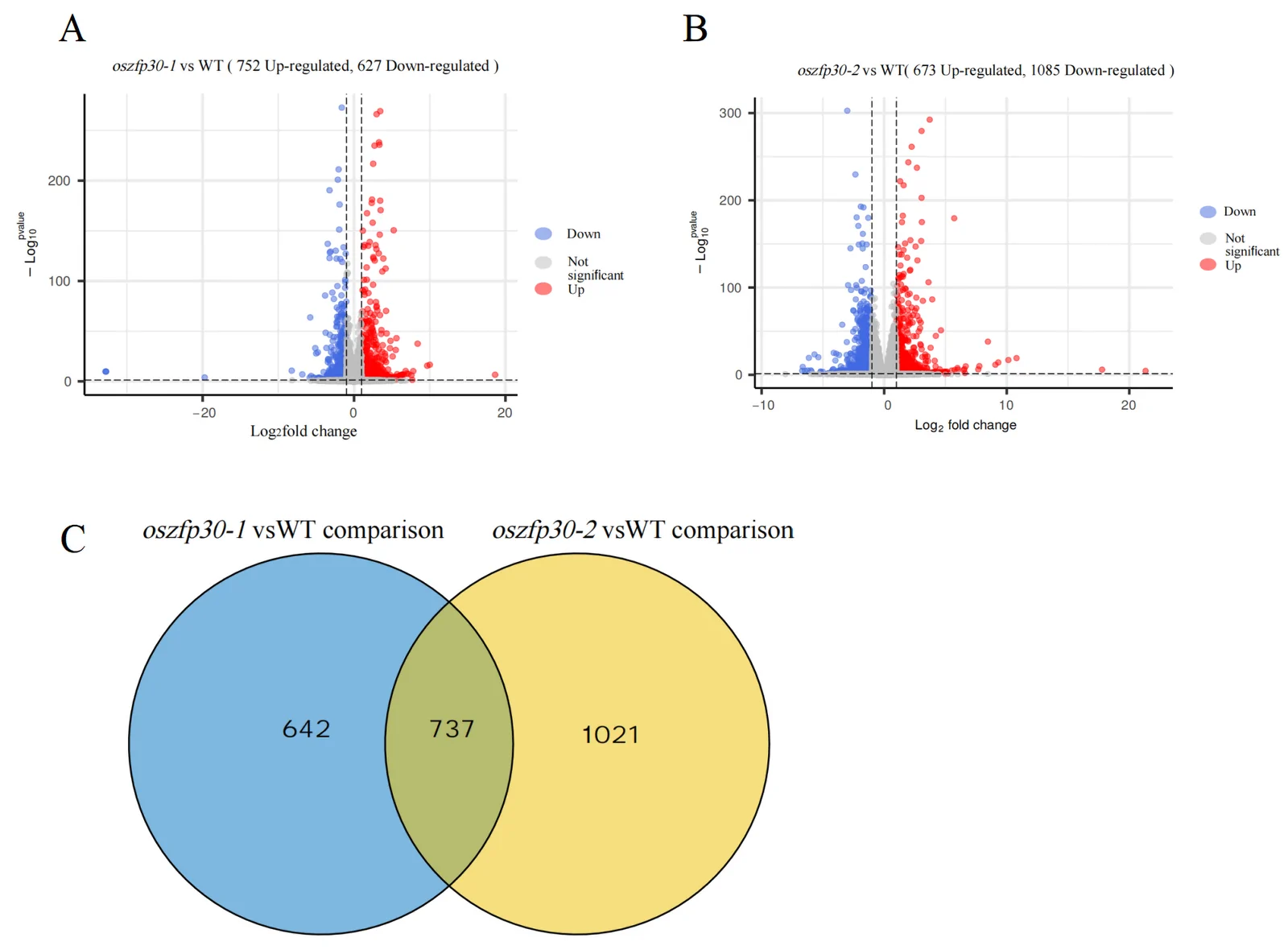
Identification and screening of DEGs. (**A**). Volcano plot of DEGs for the *oszfp30-1* vs. WT comparison group; (**B**). Volcano plot of DEGs for the *oszfp30-2* vs. WT comparison group; (**C**). Venn diagram of DEGs.

We obtained a total of 37,600 genes via transcriptome sequencing. In the *oszfp30-1* vs. WT comparison group, there were a total of 1379 DEGs, including 752 upregulated DEGs and 627 downregulated DEGs (Figure 4A). In the *oszfp30-2* vs. WT comparison group, there were a total of 1758 DEGs, including 673 upregulated DEGs and 1085 downregulated DEGs (Figure 4B). In order to identify common DEGs, we conducted an intersection comparison of the DEGs between two comparison groups. According to the Venn diagram, 876 common DEGs were identified between the two comparison groups (Figure 4C). These common DEGs may be genes associated with the *OsZFP30* mutation and warrant further in-depth research to determine whether they are directly regulated by *OsZFP30*.

### 3.6. KEGG Analysis of DEGs

**Figure 5:**
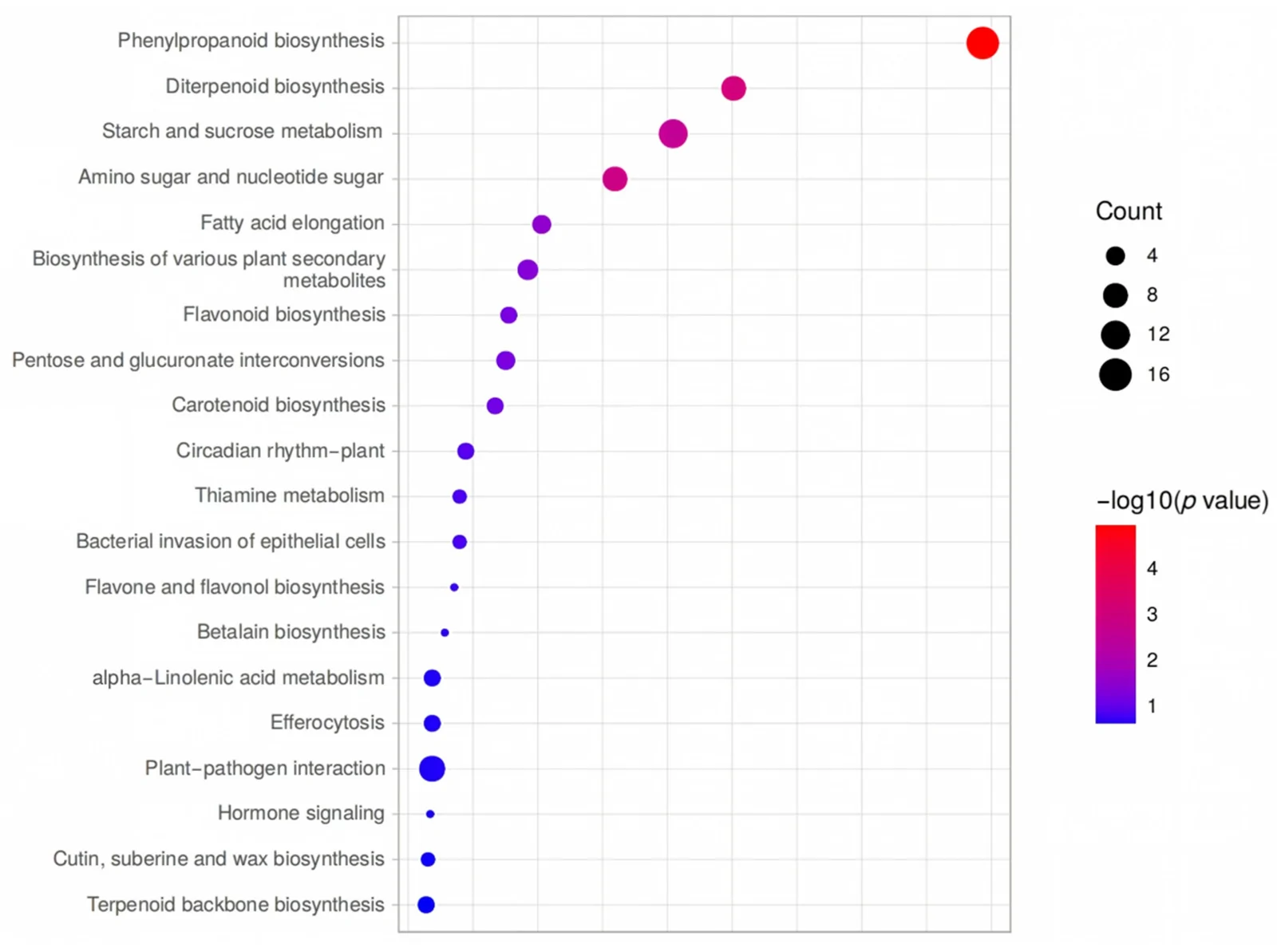
KEGG pathway diagram of common DEGs.

To explore the metabolic pathways and key genes associated with *OsZFP30*, 737 common DEGs were subjected to KEGG enrichment analysis. These DEGs were primarily enriched in diterpenoid biosynthesis, phenylpropanoid biosynthesis, starch and sucrose metabolism, and amino sugar and nucleotide sugar metabolism (Figure 5). In the diterpenoid biosynthesis pathway, seven genes were identified as key genes involved in gibberellin synthesis, including two *GA20ox*, two *GA3ox*, and three *GA2ox* (Table 3). In the phenylpropane biosynthesis pathway, eight genes were identified as key genes involved in *CCR*: three *CAD*, two *CCoAOMT*, and one *COMT* (Table 3). In the starch and sucrose metabolism pathway, six genes are key genes for cellulose synthesis, including four *OsSUS* and two *UGPase* (Table 3). In the amino sugar and nucleotide sugar metabolism pathway, six genes are key genes for hemicellulose synthesis, including five *UXS* and one *IRX10* (Table 3).

### 3.7. Statistical Analysis of Metabolomic Sequencing Data

**Figure 6:**
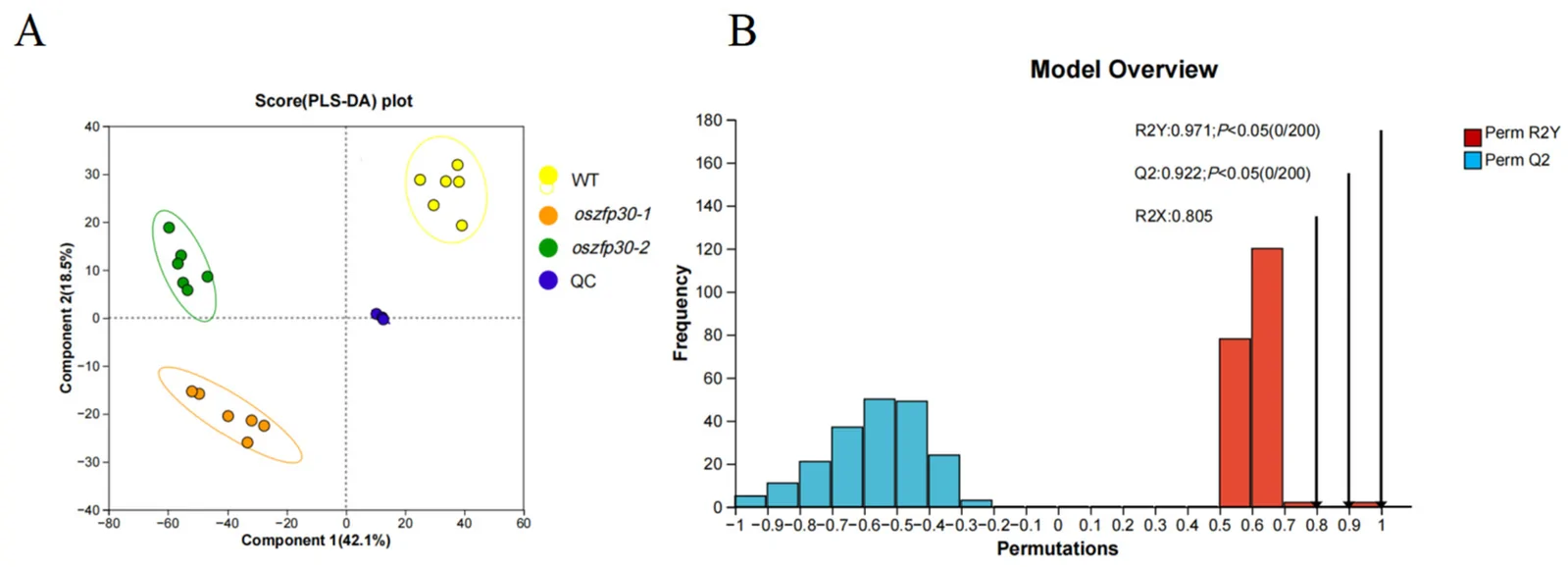
PLS-DA for metabolome data. (**A**). PLS-DA score plot. (**B**). PLS-DA permutation test.

To evaluate the differences in metabolites between the transgenic lines and the wild type, this study employed widely targeted metabolomics technology and detected metabolite components in the samples via the UPLC-ESI-MS/MS platform in multiple reaction monitoring (MRM) mode. PLS-DA was performed on the metabolomics data, and the PLS-DA score plot (Figure 6A) showed that the three sample groups could be clearly distinguished, with tight clustering within each group, indicating significant differences in their metabolic profiles. According to the PLS-DA permutation test (Table S1), the R^2^X value of the established model was 0.805, the R^2^Y value was 0.971, and the Q^2^ value was 0.922, indicating that the model exhibited a good fit and strong predictive ability (Figure 6B).

### 3.8. Identification of Differential Metabolites

**Figure 7:**
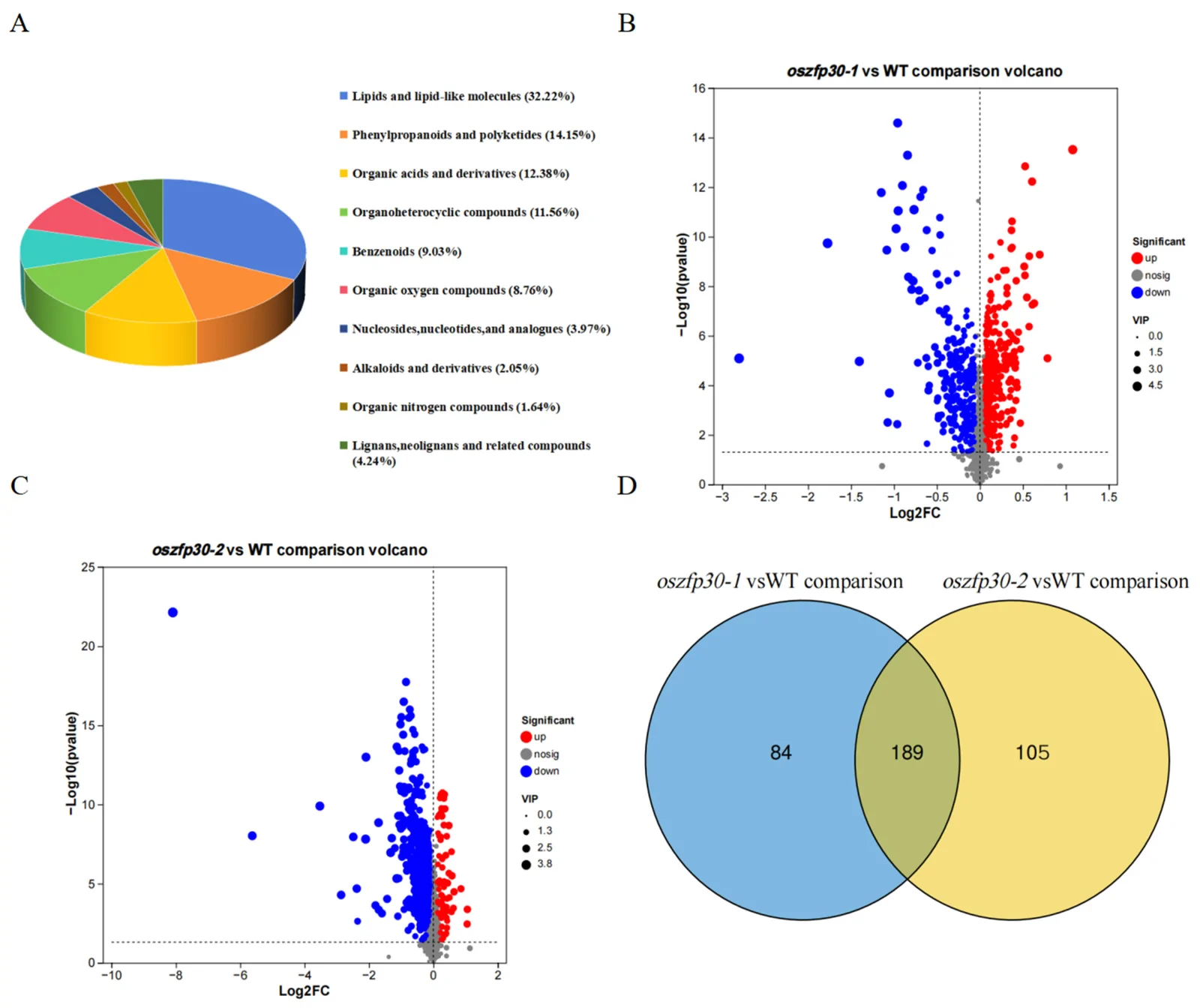
Identification of DEMs. (**A**). Circular diagram of metabolite classification. (**B**). Volcano plot of DEMs for the *oszfp30-1* vs. WT comparison group. (**C**). Volcano plot of DEMs for the *oszfp30-2* vs. WT comparison group. (**D**). Venn diagram of DEMs.

The qualitative analysis results showed that a total of 1139 metabolites were identified in the three samples, mainly lipids and lipid-like molecules (32.22%), phenylpropanoids and polyketides (14.15%), organic acids and derivatives (12.38%), organoheterocyclic compounds (11.56%), benzenoids (9.03%), organic oxygen compounds (8.76%), nucleosides, nucleotides, and analogs (3.97%), alkaloids and derivatives (2.05%), organic nitrogen compounds (1.64%), and lignans, neolignans, and related compounds (4.24%) (Figure 7A). Differentially expressed metabolites (DEMs) were then screened. In the *oszfp30-1* vs. WT comparison group, a total of 273 DEMs were identified, of which 106 were upregulated and 167 were downregulated (Figure 7B). In the *oszfp30-2* vs. WT comparison group, a total of 294 DEMs were identified, of which 126 were upregulated and 168 were downregulated (Figure 7C). According to the Venn diagram, 189 common DEMs were identified between the two comparison groups (Figure 7D). These common DEMs may be metabolites associated with the *OsZFP30* mutation and warrant further investigation.

### 3.9. KEGG Enrichment Analysis of Differential Metabolites

**Figure 8:**
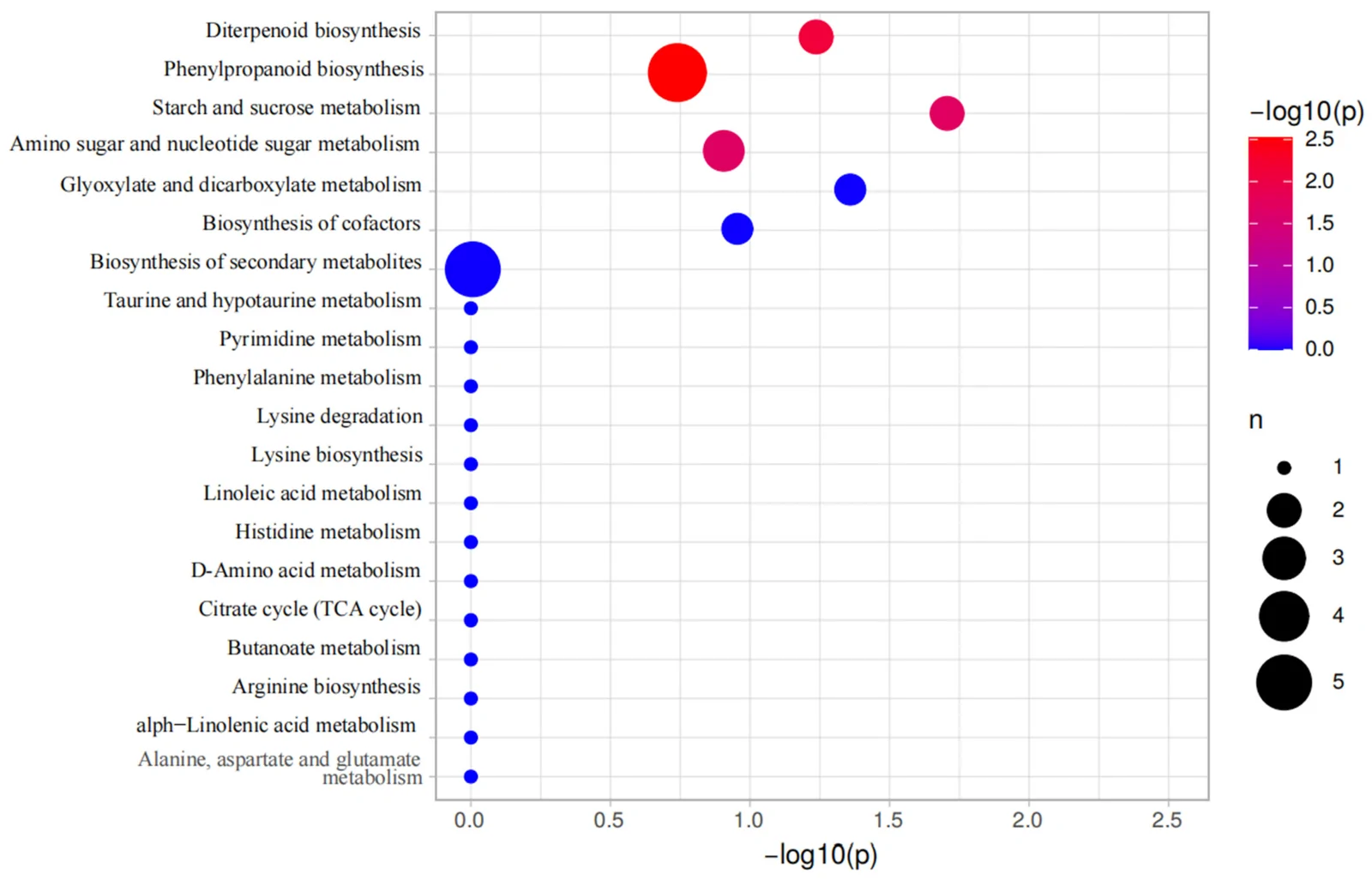
KEGG enrichment analysis of DEMs.

To further explore the biological functions of the DEMs, we used the KEGG database to annotate and enrich the selected DEMs through pathway analysis. These DEMs were mainly enriched in diterpenoid biosynthesis, phenylpropanoid biosynthesis, amino sugar and nucleotide sugar metabolism, starch and sucrose metabolism pathways (Figure 8). In the diterpenoid biosynthesis pathway, five metabolites were identified as intermediate products of GA synthesis (Table 4). In the phenylpropanoid biosynthesis pathway, five metabolites were identified as intermediate products of lignin synthesis (Table 4). In the starch and sucrose metabolism pathway, two metabolites were identified as intermediate products of cellulose synthesis (Table 4). In the amino sugar and nucleotide sugar metabolism pathway, three metabolites were identified as intermediate products of hemicellulose synthesis (Table 4).

### 3.10. qRT-PCR Verification of Differential Gene Expression Levels

**Figure 9:**
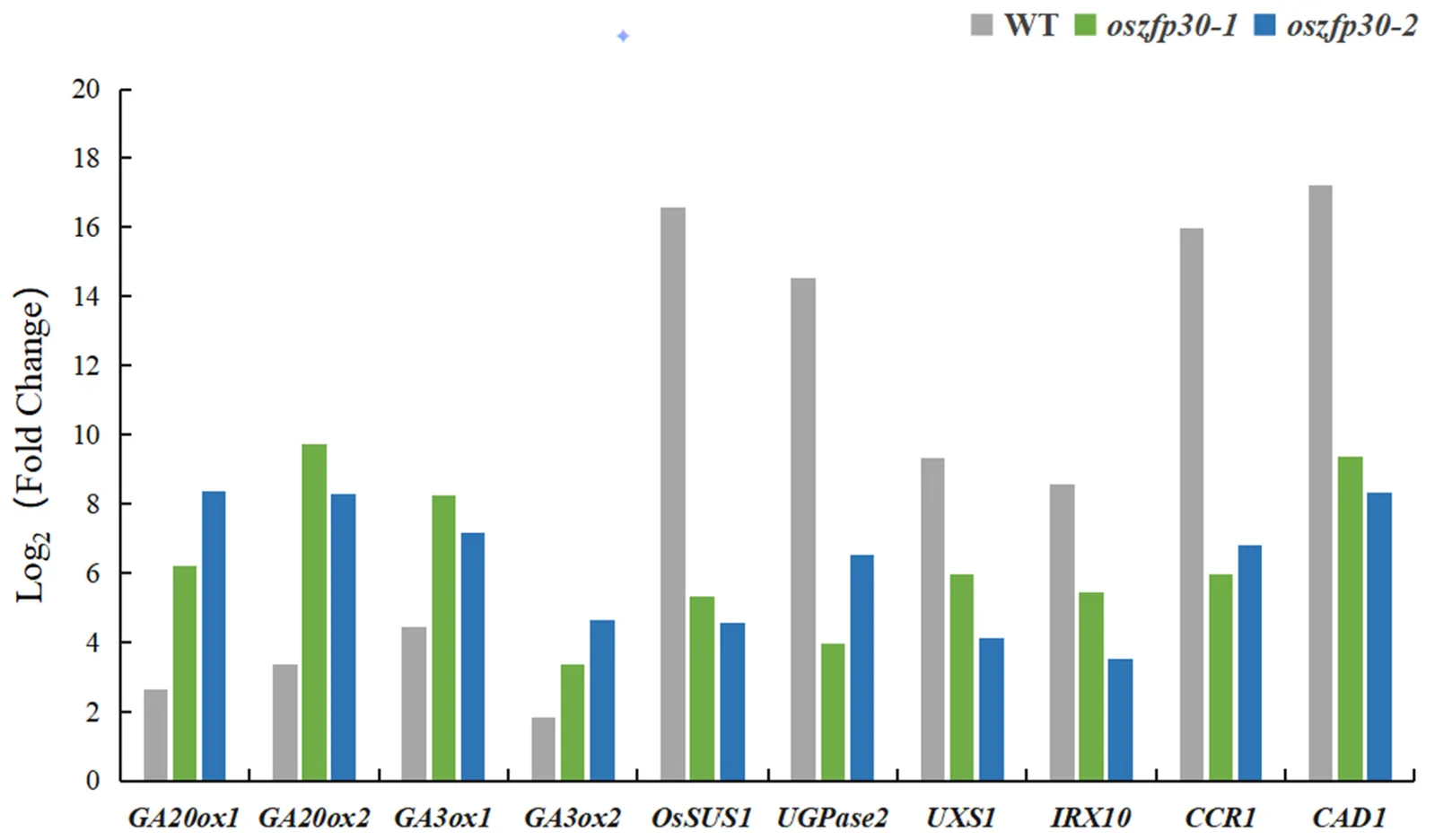
qRT-PCR verification of DEGs.

To verify the accuracy of transcriptome data, we performed qRT-PCR analysis on RNA samples used for transcriptome analysis. The results showed that the expression levels of *GA20ox1*, *GA20ox2*, *GA3ox1*, and *GA3ox2* in WT were lower than those in *oszfp30-1* and *oszfp30-2*, while the expression levels of *OsSUS1*, *UGPase2*, *UXS1*, *IRX10*, *CCR1*, and *CAD1* in WT were higher than those in (*oszfp30-1* and *oszfp30-2*) (Figure 9). These findings demonstrated that the expression patterns of these candidate genes in qRT-PCR were consistent with those observed in transcriptome data, indicating the accuracy of our transcriptome data.

## 4. Discussion

Rice straw is an unconventional roughage resource with great development potential in China, and it can serve as a high-quality roughage supplement for ruminant production [30]. High-biomass rice straw can provide more feed raw materials, effectively compensating for the low forage yield of low-biomass rice straw and significantly enhancing the forage production efficiency of paddy fields. Lignin, cellulose, and hemicellulose are the main structural components of the straw cell wall and are also key anti-nutritional factors that limit the feeding value and digestibility of rice straw [31]. Reducing their content has significant positive implications for the efficient utilization of rice straw as feed. Lower levels of structural fiber components can significantly improve the feeding quality and palatability of rice straw [32]. Lignin is chemically stable and resistant to degradation by rumen microorganisms in ruminants [33]. It also encapsulates cellulose and hemicellulose, hindering the release of carbohydrates, which is the core factor limiting straw digestibility. Reducing the lignin content in rice straw can effectively break down the dense cell wall structure, reduce straw hardness and roughness, and enhance its palatability [34]. At the same time, decreased cellulose and hemicellulose contents can reduce the overall fiber proportion of the straw, improve its relative nutritional value, and result in higher available nutrient content per unit mass, thereby improving overall feeding quality [35]. In this study, compared with the wild type, the transgenic lines had lower lignin, cellulose, and hemicellulose contents, indicating their great potential for feed utilization. It should be noted that the present study evaluated forage quality based on chemical composition (cellulose, hemicellulose, lignin, crude protein, and crude fat) rather than direct digestibility or feeding trials. The compositional changes observed suggest improved forage potential, but in vitro dry matter digestibility (IVDMD) or in vivo ruminal degradation experiments are needed to confirm the actual feeding value.

To analyze why *oszfp30-1* and *oszfp30-2* had higher forage quality, we conducted transcriptomic and metabolomic analyses on these lines and the wild type. The results showed that the metabolic pathways significantly enriched at both the transcriptional and metabolic levels were diterpenoid biosynthesis, phenylpropanoid biosynthesis, and starch and sucrose metabolism. Therefore, the differentially expressed genes in these three metabolic pathways may be closely related to the high forage quality of the transgenic lines. Gibberellin (GA) is a type of diterpenoid plant hormone, and GA treatment can significantly enhance the forage value of rice straw and increase panicle weight [16,36,37]. GA20-oxidase and GA3-oxidase are key enzymes in the final step of the GA biosynthesis pathway. Specifically, GA20ox converts GA12 and GA53 into GA9 and GA20, while GA3ox further converts GA9 and GA20 into GA1 and GA4 [38,39]. GA2 oxidase is a key enzyme in the degradation process of GA; it can break down and inactivate GAs, their precursors, and other intermediate products with biological activity in plants, thereby maintaining the balance between GAs and intermediates with biological activity in plants [40]. The absence of *GA20ox* and *GA3ox* reduces the amount of bioactive GA synthesized in rice [41]. Similarly, overexpressing GA2ox enzymes degrades bioactive GA in rice, leading to a dwarf phenotype [42]. In this study, the upregulated expression of GA20ox and GA3ox genes, coupled with the downregulated expression of the GA2ox gene, led to an increase in GA content in *oszfp30-1* and *oszfp30-2*.

The fibrous substances of rice straw mainly include cellulose, hemicellulose, and lignin, and within these cellulose accounts for 25–45%, hemicellulose accounts for 25–30%, and lignin accounts for 10–15% [43]. Cellulose molecules are made up of β -1,4-glucan chains, which are the main components of cell walls and form complex polymer networks with hemicellulose and pectin [44]. The core key enzyme for plant cellulose synthesis is cellulose synthase (CESA), which directly catalyzes the polymerization of UDP glucose to form cellulose chains [45]. Uridine diphosphate glucose pyrophosphate synthase (UGPase) and sucrose synthase (SUS) are important precursor synthases that provide the key substrate UDP glucose [46]. The mutation of the Brittle culm19 gene encoding the cellulose synthase subunit CESA4 in rice resulted in a significant decrease in the cellulose, hemicellulose, and lignin contents in the plant, but did not affect plant growth and rice yield [47]. SUS is encoded by a multigene family, which is divided into three types: SUS I, SUS II, and SUS III. It is involved in processes such as starch biosynthesis, cellulose synthesis, and sugar metabolism [48]. Transgenic rice plants overexpressing the *OsSUS* gene showed a significant increase in grain weight and cell wall cellulose content [49,50]. Overexpression of *GhUGP* gene in *Arabidopsis* resulted in an increase in cellulose content but no increase in lignin content in transgenic Arabidopsis plants [51]. The *SMS1* gene in rice encodes a UGPase enzyme, and its sms1 mutant exhibits defects in cell wall structure [52]. In this study, we speculate that the downregulation of the genes encoding SUS and UGPase in *oszfp30-1* and *oszfp30-2* leads to a decrease in UDP-glucose content, which in turn results in reduced cellulose content in the mutants.

Hemicellulose is a heteropolysaccharide whose primary biological function is to crosslink with cellulose and lignin, embedding in cellulose crystals [43]. In the primary cell wall, hemicellulose is mainly composed of xyloglucan and galactomannan, while in the secondary cell wall, xyloglucan becomes the most predominant hemicellulose polysaccharide [53]. Xylan synthase is a key enzyme in the biosynthesis process of hemicellulose, which includes IRX9, IRX10, and IRX14. The *irx9*, *irx10*, and *irx14* mutants of *Arabidopsis* all exhibit a typical “irregular xylem” (IRX) phenotype, including ductal cell collapse, plant dwarfism, and significant thinning of secondary cell wall thickness [54]. In addition, the hemicellulose content of these mutants is lower than that of the wild type. *OsIRX10* is involved in the extension of the main chain of the rice hemicellulose xylan, and it was found that the *osirx10* mutant exhibited stem brittleness with significantly reduced hemicellulose and cellulose content [55]. The biosynthesis of xylan requires a stable and sufficient supply of UDP-xylose, which is primarily catalyzed by UDP-glucuronic acid decarboxylase (UXS) from UDP-GlcA. Compared to the control, the transgenic *A. thaliana* plants with downregulated *UXS* expression showed reduced levels of xylose and cellulose [56]. Rice *FC18* encodes *OsUXS3*, and the rice *FC18* mutant had reduced xylose levels and decreased xylan content [57]. In addition, the *fc18* mutant showed a decrease in cellulose content, a decrease in crystallinity, and an increase in biomass digestibility. In this study, we hypothesize that the downregulation of genes encoding UXS and IRX10 leads to decreased levels of UDP-xylose and 1,4-β-D-xylan, ultimately resulting in reduced hemicellulose content in *oszfp30-1* and *oszfp30-2*.

Lignin is primarily deposited in the secondary cell wall and consists of three monomers: hydroxyphenyl lignin (H), guaiacyl lignin (G), and syringyl lignin (S). It is crucial for the integrity of the cell wall and the strength and toughness of the stem [58]. Genetic modification can reduce lignin content in plants and/or alter lignin composition to improve the digestibility of forage, but such genetic modification often comes with adverse traits [59]. The biosynthetic pathway of lignin is relatively complex, requiring the involvement of multiple enzymes. The overexpression of rice *OsCCR1*, *OsCAD2 OsCCoAOMT1*, and *OsCOMT* genes resulted in a significant accumulation of lignin content in mutant plants [60,61]. The downregulation of caffeic acid 3-O-methyltransferase (*COMT*) and caffeoyl CoA 3-O-methyltransferase (*CCoAOMT*) in alfalfa resulted in a reduction in lignin content by up to 30% and 18%, respectively [62]. The silencing of *COMT* and *CCoAOMT* reduces lignin content by decreasing the ratios of S monomers and G monomers, respectively, and both enhance dry matter digestibility [62]. In tobacco transgenic lines, the activities of *CAD* and *CCR* were simultaneously downregulated, leading to a significant decrease in lignin content, but without a significant impact on plant development [63]. The silencing of *CAD* reduced the S-lignin content in alfalfa, but had no effect on the total lignin content [64]. The irx4 mutant with a defect in the CCR gene in *A. thaliana* had a lignin content 50% lower than that of wild-type plants, while the cellulose and hemicellulose contents remained unchanged [65]. In this study, we speculate that the downregulation of *CCR*, *CAD*, *CCoAOMT*, and *COMT* genes in *oszfp30-1* and *oszfp30-2* leads to decreased levels of metabolic intermediates in the lignin synthesis pathway, ultimately resulting in reduced lignin content in these lines. It should be noted that the present transcriptomic and metabolomic data demonstrate associations rather than direct regulatory relationships. Further experiments via means such as ChIP-seq, yeast one-hybrid (Y1H), and dual-luciferase reporter assays are needed to confirm whether OsZFP30 directly binds to the promoters of these candidate genes.

## 5. Conclusions

In summary, this study first demonstrated through phenotypic and physiological analyses that the transgenic rice lines exhibited improved nutritional and cell wall compositional traits, including higher biomass, crude protein, and crude fat, and lower cellulose, lignin and hemicellulose contents. We then analyzed the reasons for this improvement through transcriptomic and metabolomic analyses. The improvement was mainly attributed to increased gibberellin content and decreased cellulose, hemicellulose, and lignin contents, associated with altered expression levels of key genes and metabolites involved in gibberellin, cellulose, hemicellulose, and lignin synthesis pathways. The key genes identified in this study lay a foundation for further functional validation of the *OsZFP30* transcription factor. Future studies should include in vitro dry matter digestibility (IVDMD) or ruminal degradation experiments to directly confirm the forage quality improvement suggested by these compositional changes.

## Figures and Tables

**Table 1:** Comparison of agronomic traits between lines and wild-type rice.

Material	Plant Height (cm)	Number of Tillers (Plant^−1^)	Above-Ground Biomass (g)
WT	95.5 ± 3.2 b	12.3 ± 1.1	29.12 ± 0.96 b
*Oszfp30-1*	99.8 ± 3.0 a	12.5 ± 1.0	35.56 ± 0.80 a
*Oszfp30-2*	99.2 ± 3.1 a	12.1 ± 1.2	34.21 ± 0.77 a

Note: Different lowercase letters within the same column indicate significant differences (*p* < 0.05). Data are presented as mean ± standard deviation.

**Table 2:** Transcriptome sequencing data quality assessment.

Sample	Total Reads (M)	Total Bases (G)	GC Content (%)	Q20 (%)	Q30 (%)	Mapped Reads (%)
WT-1	64.93	9.74	48.63	99.00	96.41	95.06
WT-2	64.12	9.62	48.70	99.02	96.47	95.36
WT-3	75.02	11.25	49.22	99.04	96.56	95.45
*oszfp30-1-1*	63.68	9.55	49.41	99.01	96.45	95.06
*oszfp30-1-2*	60.56	9.08	48.90	99.01	96.44	95.42
*oszfp30-1-3*	58.85	8.83	49.50	98.95	96.28	96.17
*oszfp30-2-1*	58.98	8.85	48.59	98.92	96.12	96.20
*oszfp30-2-2*	65.78	9.87	48.80	98.97	96.34	95.44
*oszfp30* *-2-3*	56.91	8.54	48.63	99.00	96.38	95.05

**Table 3:** Key DEGs related to rice straw forage quality.

KEGG Name	Gene Name	Gene Number	Expression Pattern
Diterpenoid biosynthesis	*GA20ox1*	LOC4325003	Up
	*GA20ox2*	LOC4334841	Up
	*GA3ox1*	LOC4323864	Up
	*GA3ox2*	LOC4337968	Up
	*GA2ox1*	LOC4325145	Down
	*GA2ox2*	LOC4325537	Down
	*GA2ox3*	LOC4335758	Down
Starch and sucrose metabolism	*OsSUS1*	LOC4331252	Down
	*OsSUS2*	LOC4332788	Down
	*OsSUS3*	LOC4333062	Down
	*OsSUS4*	LOC4335303	Down
	*UGPase1*	LOC4326201	Down
	*UGPase2*	LOC4328091	Down
Amino sugar and nucleotide sugar metabolism	*UXS1*	LOC4327295	Down
	*UXS2*	LOC4327367	Down
	*UXS3*	LOC4332424	Down
	*UXS4*	LOC4332441	Down
	*UXS5*	LOC4338546	Down
	*IRX10*	LOC107276048	Down
Phenylpropanoid biosynthesis	*CCR1*	LOC107278880	Down
	*CCR2*	LOC4323945	Down
	*CAD1*	LOC4328552	Down
	*CAD2*	LOC4335223	Down
	*CAD3*	LOC4336968	Down
	*CCoAOMT1*	LOC4340240	Down
	*CCoAOMT2*	LOC4347397	Down
	*COMT1*	LOC4344702	Down

**Table 4:** Key DEMs related to rice straw forage quality.

KEGG Name	Index	Name	Expression Pattern	Entry
Diterpenoid biosynthesis	MW0139382	Gibberellin A9	Up	C11863
	MW0112732	Gibberellin A4	Up	C11864
	MW0108646	Gibberellin A34-catabolite	Down	C11869
	MW0003179	Gibberellin A12	Down	C11857
	MW0113755	Gibberellin A53	Down	C06094
Starch and sucrose metabolism	MW0138536	UDPglucose	Down	C00029
	MW0119612	Sucrose	Up	C00089
Amino sugar and nucleotide sugar metabolism	MW0141380	UDP-xylose	Down	C00190
	MW0049085	1,4-beta-D-Xylan	Down	C02352
	MW0010721	Wood sugar	Down	C00181
Phenylpropanoid biosynthesis	MW0011597	Caffeic aldehyde	Down	C10945
	MW0114048	Sinapyl alcohol	Down	C02325
	MW0014155	Coniferyl aldehyde	Down	C02666
	MW0012936	Coniferyl alcohol	Down	C00590
	MW0110241	Sinapoyl aldehyde	Down	C05610

## Data Availability

All data are presented in this article.

## Supplementary Materials

The following supporting information can be downloaded at: https://www.mdpi.com/article/10.3390/genes17091086/s1. Figure S1. *OsZFP30* gene sequence; Figure S2. Knockout primer of *OsZFP30*; Figure S3. Dual target sequence and two *Oszfp30* mutants; Table S1. Sample plsda origin.
