# Supplementary material for: Analysis of the Mechanisms of Rice oszfp30 Mutant in Improving Forage-Quality-Related Traits Based on Transcriptome and Metabolome Analyses
Source: Genes (Basel). 2026 Sep 9;17(9):1086. doi: 10.3390/genes17091086 (PMC13606066; doi:10.3390/genes17091086)
Supplement: Supplementary file 1 [file genes-17-01086-s001.zip › Supplementary/oszfp30-CRISPR.2.pdf]

ATGAACATCCAGAGGAAGCCAGGAGACTGGAACTGCAAATCGTGCCAGCATCTCAAC
TTCAGCCGCCGGGACTACTGCCAGCGCTGCCATACCCCACGCCAGGACCTGCCGCTTG
GCGATGGTTATGTCCCAGGTGGTGTGCTGACCTCCCTGGACATTCGCCCGGGCGACTG
GTACTGCAACTGCGGCTATCACAACTTTGCTAGCCGAGCAAGCTGCTTCAAATGTGGC
GCCATTGTGAAGGACCTTCCAGCAGGCCAAGGTGGTGGTGTTGCCAACGGTGACTTTG
CCCGTGCCCTCGACAGCAGCGCAGTTCGTGCTGGGTGGAAGGCAGGCGACTGGATTT
GCACAAGGCCTGGTTGCAACGTCCACAACTTTGCAAGTAGGATTGAGTGCTATAGGTG
CAATGCACCTAGGGAAGCAGGTAATGTGAAGTAA

Figure S1 *OsZFP30* gene sequence

gRT1: GCGATGGTTATGTCCCAGGgttttagagctagaaat
OsU6aT1: CCTGGGACATAACCATCGCCggcagccaagccagca

gRT2: ACAGCAGCGCAGTTCGTGCTgttttagagctagaaat
OsU6bT2: AGCACGAACTGCGCTGCTGTCaacacaagcggcagc

Figure S2 Knockout primer of *OsZFP30*

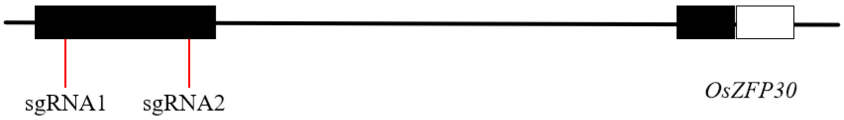

	Target sitet1 PAM	Target sitet2 PAM
WT	GGCGATGGTT-TGTCCCAGGTGG---	ACAGCAGCGCAGTTC-GTGCTGGG
oszfp30-1	GGCGATGGTTATGTCCCAGGTGG---	ACAGCAGCGCAGTTC-GTGCTGGG
oszfp30-2	GGCGATGGTT-TGTCCCAGGTGG---	ACAGCAGCGCAGTTCTGTGCTGGG

Figure S3 Dual target sequence and two Oszfp30 mutants
